# Comparison: Flu Prescription Sales Data from a Retail Pharmacy in the US with Google Flu Trends and US ILINet (CDC) Data as Flu Activity Indicator

**DOI:** 10.1371/journal.pone.0043611

**Published:** 2012-08-30

**Authors:** Avinash Patwardhan, Robert Bilkovski

**Affiliations:** Walgreen Co., Deerfield, Illinois, United States of America; Harvard School of Public Health, United States of America

## Abstract

The potential threat of bioterrorism along with the emergence of new or existing drug resistant strains of influenza virus, added to expanded global travel, have increased vulnerability to epidemics or pandemics and their aftermath. The same factors have also precipitated urgency for having better, faster, sensitive, and reliable syndromic surveillance systems. Prescription sales data can provide surrogate information about the development of infectious diseases and therefore serve as a useful tool in syndromic surveillance. This study compared prescription sales data from a large drug retailing pharmacy chain in the United States with Google Flu trends surveillance system data as a flu activity indicator. It was found that the two were highly correlated. The correlation coefficient (Pearson ‘r’) for five years' aggregate data (2007–2011) was 0.92 (95% CI, 0.90–0.94). The correlation coefficients for each of the five years between 2007 and 2011 were 0.85, 0.92, 0.91, 0.88, and 0.87 respectively. Additionally, prescription sales data from the same large drug retailing pharmacy chain in the United States were also compared with US Outpatient Influenza-like Illness Surveillance Network (ILINet) data for 2007 by Centers for Disease Control and Prevention (CDC). The correlation coefficient (Pearson ‘r’) was 0.97 (95% CI, 0.95–0.98).

## Introduction

Detection and tracking of infectious diseases, that affect populations rather than individuals, has always been a high priority in the minds of the public health officials. However, the innovations and implementation of systems towards that effect became accelerated in the United States since the 2001 anthrax outbreak [1]. Nonetheless, the role and the purpose of such systems, also known as clinical surveillance systems or syndromic surveillance systems (SSS) is not restricted to or applicable to only the threat of terrorism. High mobility of people across large distances, increasing adaptation of bacteria and viruses to become drug resistant, and increasing general health care costs have made the need for robust, efficient, sensitive, and reliable SSSs that can monitor diseases and their outbreaks dynamically, urgent.

Centers for Disease Control and Prevention (CDC) defines syndromic surveillance as “an investigational approach where health department staff, assisted by automated data acquisition and generation of statistical alerts, monitor disease indicators in real-time or near real-time to detect outbreaks of disease earlier than would otherwise be possible with traditional public health methods” [2]. Similarly, “the fundamental objective of syndromic surveillance is to identify illness clusters early, before diagnoses are confirmed and reported to public health agencies, and to mobilize a rapid response, thereby reducing morbidity and mortality” [1].

All SSSs depend on large volumes of data. Broadly, data sources are classified as traditional and non-traditional or sometimes as clinical and non-clinical. Pavlin et al. enumerate 21 types of data sources that can be used for the purpose [3]. Data from pharmacy sales, calls to emergency services, internet hits or chatters for medical information, work or school attendance records are a few examples of non-traditional data sources and serve as surrogate indicators of disease activity [1].

Nature of data inputs, efficiency of analysis, speed of response [4], confidentiality [5], and sensitivity and specificity [6] are a few criteria that determine how good an SSS is.

The International Society for Disease Surveillance [7] conducted a survey (2007–2008) of public health officials in the United States to determine what data sources were commonly used for surveillance. 84% used emergency department visits, 49% used outpatient clinic visits, 44% used over the counter drugs (OTC), 35% used school absenteeism, and only 7% used pharmacy prescription sales data [8]. At the national syndromic surveillance conference in 2003 there were only two papers that discussed prescription sales data [9]. For the same conference in 2004, there was only one [10].

There could be many possible reasons for relatively low use of prescription sales data for syndromic surveillance. Non specificity, reflection of symptoms rather than lab diagnosis, and influence by market promotions could make their use less attractive [3]. Less availability in the public domain could be another [11]. Lack of sufficient public-private partnerships to harness them could also contribute to their uncommon use. Likewise, the private owners of the prescription sales data may find data extraction and analysis hard, confidentiality issues challenging, resource constraints significant, financial incentives lacking, and data storage difficult [12].

Despite practical barriers, there have been many reports, within the US as well as internationally, suggesting effective use of prescription sales data in syndromic surveillance. The New York State Department of Health (NYSDOH) has used prescription sales data for the Medicaid population successfully in syndromic surveillance [10]. In October 2009 Rhode Island launched a statewide system for tracking Swine Flu using prescription data. In their model, pharmacies like Walgreens, CVS, Rite Aid, and Stop & Shop provided de-identified data on prescription of Tamiflu and other antiviral drugs [13]. Walter Reed Army Institute of Research at Veteran's Affairs have analyzed prescribing patterns of psychotropic medications to monitor changes in a community's behavioral health status [14]. Copeland examined two models of the use of prescription sales data collected and analyzed by IMS Health [15] to conclude that “Prescription data for Tamiflu appear to reflect patterns of influenza as reported by CDC, across time both nationally and regionally” [16]. A Japanese report found ‘potential for monitoring influenza activity and for providing early detection of infectious disease outbreaks’ in an automatic surveillance system, monitoring prescription drug purchases of Oseltamivir, Zanamivir, and Laninamivir [17]. A quarterly review (2010) published by National Institute of Science & Technology Policy of Japan states that prescription drugs surveillance serves as a very useful tool in influenza monitoring in Japan [18]. Syndromic surveillance using medications sales has been found useful in France [19]. A Dutch study also found value in the use of prescription sales data in syndromic surveillance [20].

Community pharmacies have started playing an increasingly important role in immunization services [21]. Therefore it is possible that they might also be able to contribute to syndromic surveillance of infectious diseases. In line with this thinking, we desired to determine if the use of prescription sales data in syndromic surveillance could be revalidated by comparing them with the data from an established surveillance system.

Recently syndromic surveillance systems based on internet searches or communications (Google, Twitter) have become popular. In general scholars agree on the utility of these systems [22] [23] [24] [25] [26] [27]. Nonetheless, concerns also have been raised about the limitations and inadequacies of those methods [6]. Because Google data are relatively easy to access and manage, in this study we endeavored to examine if the prescription sales data from a large drug retailing pharmacy chain in the United States were comparable to Google Flu trends surveillance system data as flu activity indicator. Using traditional data source, the epidemiology and prevention branch in the influenza division at CDC performs influenza surveillance activity year round in the United States to produce FluView, a weekly influenza surveillance report [28]. Data associated with those activities are available in public domain [29]. Google has validated their data against CDC's US Outpatient Influenza-like Illness Surveillance Network (ILINet) data [22]. Therefore we also decided to compare the prescription sales data from the same large drug retailing pharmacy chain in the United States with the best available data from the ILINet.

## Methods

We extracted de-identified prescription sales data (script count) from the proprietary pharmacy computer system of a large drug retailing pharmacy chain in the United States with more than 8000 locations across all 50 states. These data, de-identified prior to our analysis, were extracted by our IT Colleagues who manage the enterprise data warehouse for the drug retailing pharmacy chain. All customers of the said drug retailing pharmacy chain are provided with and have access to the Notice of Privacy Practices which includes within it a description of how drug retailing pharmacy chain can use patient data for health care operations and research purposes. The drug retailing pharmacy chain can use patient information when it has been de-identified. This chain wide system covers prescriptions entered at all its retail as well as central stores. We included prescriptions entered into the system for a fill between January 1, 2007 and December 31, 2011 covering a period of five years. The prescriptions were written for the four drugs commonly prescribed for the treatment of influenza namely: Amantadine, Oseltamivir, Rimantadine, and Zanamivir. We counted all the National Drug Codes (NDCs) associated with each drug; 22 for Amantadine, 16 for Oseltamivir, 5 for Rimantadine, and 3 for Zanamivir. We counted all the prescriptions brought for a fill and therefore entered into the system, regardless of whether they were actually sold and picked up by a patient. Occasionally when a plan denies approval a patient does not pick up medications. The prescription counts were bucketed in a weekly group that began on Sunday and ended on Saturday- exactly in the same way as the comparison group (Google) had arranged. Google data rounds off the days around the yearend in a way slightly different from ours and we made sure that we altered our groups at those points to align with Google data. For example, in December 2007, Google count on January 6, 2008 would include influenza like illness (ILI) covering December 30 through January 5, 2008 in a single group, whereas in our database, in the same period, those numbers would appear in two groups, one counting prescriptions for December 30 and 31, 2007, and another for January 1, 2008 through January 5, 2008. Additionally, we also counted the total number of prescriptions (regardless of drug class or name) entered for a fill in our system for every weekly group in our study period. Then we added the counts for all the four drugs per week into a single group. Then we generated a per 100,000 scripts number for each aggregated count as above, using the total number of prescriptions entered for a fill in our system for the respective week in the study period.

Google Flu trends surveillance system data are available in the public domain and can be freely downloaded. We acquired those data on February 10, 2012 in an Excel (2010) Workbook. We selected data between January 1, 2007 and December 31, 2011 for our comparison. We included only the national aggregate counts (United_States). These numbers represented the estimates of the ILI cases per 100,000 physician visits [30]. Note that the number in each row of the Google dataset represents the total count of estimated ILI cases per 100,000 physicians visits during the week preceding the corresponding date, where the week begins on Sunday and ends on Saturday. For example, the number 2199 against January 6, 2008 means that there were 2199 ILI cases per 100,000 physicians visits in the United States, for the days December 30, 2007 through January 5, 2008.

We compared our per 100,000 influenza scripts number for each aggregated count with the corresponding Google ILI cases per 100,000 physicians visits in the United States for the study period between January 1, 2007 and December 31, 2011 using Excel (2010). We calculated Pearson product moment correlation coefficient between our data and Google data, at year to year as well as aggregate level. We used the function “CORREL” for our purposes. We also created comparable trends graphs from the five years aggregate data, after converting the counts of the influenza scripts and the counts of the Google estimated ILI cases to logarithmic scale. Such conversion made visualization of the data much clearer.

For comparing our prescription sales data as above with CDC influenza-like illness data, we acquired CDC data for 2007 from CDC website [29]. We downloaded the file for 2007–2008 ‘senregallregion07–08.csv’ as Excel (2010) workbook on the CDC website under U.S. Outpatient Influenza-like Illness Surveillance Network (ILINet). This file contained, as per the top row in the file, ‘Weekly Percents of Visits for Influenza-like Illness (ILI) Reported by the U.S. Outpatient Influenza-like Illness Surveillance Network (ILINet) National Summary 2007–08’. We took the % unweighted ILI for comparison rather than the % weighted ILI because CDC uses the basic of state population to calculate the latter [28]. Our prescription sales data were not sorted on the basis of state population. CDC has only published data until 2010. We looked at 2008–2009 and 2009–2010 data but did not find them sufficiently complete or clean for use. Our method for calculating the Pearson product moment correlation coefficient between our data and CDC data was the same as before.

## Results

The Pearson ‘r’ between the aggregate counts of scripts for all the four drugs commonly prescribed for influenza namely: Amantadine, Oseltamivir, Rimantadine, and Zanamivir, expressed as the influenza drugs scripts per 100,000 total scripts filled at a large drug retailing pharmacy chain in the United States and the Google estimates of the ILI cases per 100,000 physicians visits in the United States for years 2007, 2008, 2009, 2010, and 2011 were 0.85 (95% CI, 0.75–0.91), 0.92 (95% CI, 0.86–0.95), 0.91(95% CI, 0.85–0.95), 0.88 (95% CI, 0.80–0.93), and 0.87 (95% CI, 0.78–0.92). The Pearson ‘r’ for the aggregate data (2007 through 2011) comparison was 0.92 (95% CI, 0.90–0.94). The Pearson ‘r’ between the aggregate counts of scripts for all the four drugs commonly prescribed for influenza namely: Amantadine, Oseltamivir, Rimantadine, and Zanamivir, expressed as the influenza drugs scripts per 100,000 total scripts filled at a large drug retailing pharmacy chain in the United States and the CDC % unweighted ILI was 0.97 (95% CI, 0.95–0.98). See Table 1.

**Table 1:** Pearson product moment correlation coefficient (‘r’) between prescription sales data from a large drug retailing pharmacy chain in the United States and Google Flu trends data and CDC ILI data.

Prescription sales data and	Year	Pearson ‘r’	95% CI
Google Trends ILI data	2007	0.85	0.75–0.91
	2008	0.92	0.86–0.95
	2009	0.91	0.85–0.95
	2010	0.88	0.80–0.93
	2011	0.87	0.78–0.92
	Aggregate (2007–2011)	0.92	0.90–0.94
CDC % unweighted ILI data	2007	0.97	0.95–0.98

Pearson product moment correlation coefficient (‘r’) between (1) The aggregate counts of scripts for four drugs commonly prescribed for influenza namely: Amantadine, Oseltamivir, Rimantadine, and Zanamivir expressed as scripts per 100,000 total scripts and the Google trends data Influenza-like Illness (ILI) cases per 100,000 physicians visits:2007–2011, year by year and aggregate 2007–2011 and (2) The prescription sales data for 2007 from the above prescription sales data and % unweighted ILI data for 2007 from CDC's Outpatient Influenza-like Illness Surveillance Network (ILINet).

**Figure 1:**
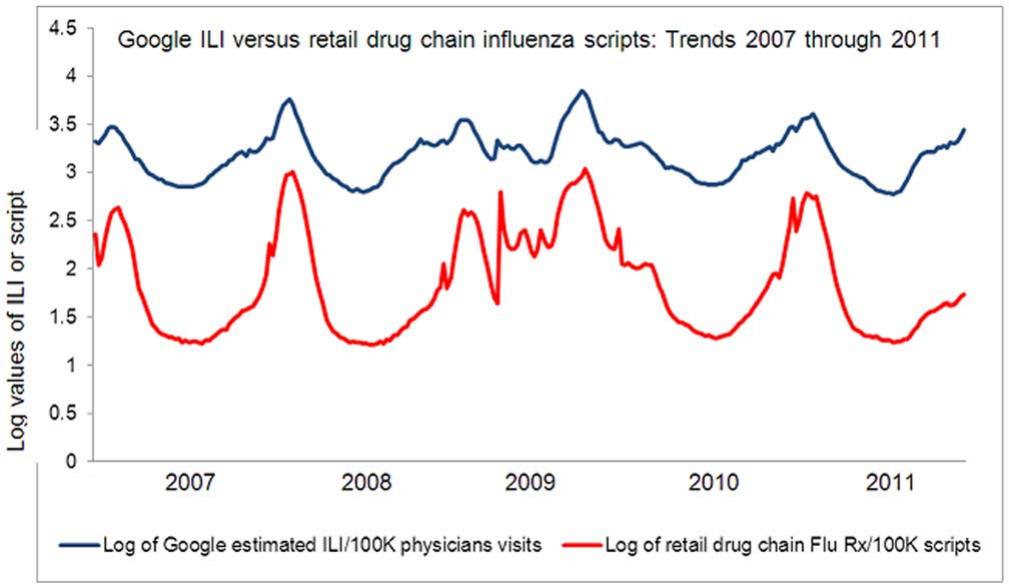
Google ILI versus retail drug chain influenza scripts: Trends: 2007 through 2011. The comparative graphic representation of the Google ILI data as cases per 100,000 physicians visits for five years (2007–2011) and aggregate counts of scripts for four drugs commonly prescribed for influenza namely: Amantadine, Oseltamivir, Rimantadine, and Zanamivir from a large drug retailing pharmacy chain in the United States expressed as scripts per 100,000 total scripts for five years (2007–2011) after log transformation.

Figure 1. depicts a clear similarity in the trends patterns created by prescription sales data from a large drug retailing pharmacy chain in the United States and Google Flu trends surveillance system data.

## Discussion

Copeland et al. say, “The prescription data are timely and enhance capabilities for quantification and localization of outbreak detection, thereby addressing the initial detection, quantification, and localization factors for successful early detection” [16]. A strong to very strong correlation between prescription sales data from a large drug retailing pharmacy chain in the United States and Google Flu trends surveillance system data for influenza and a very strong correlation between prescription sales data from the same large drug retailing pharmacy chain in the United States and CDC's ILI activities data for influenza suggests that the former can serve as a good, valid, and independent influenza activity indicator or a syndromic surveillance system.

Prescription sales data can be easily and quickly mined. It is another matter that currently there may not be well developed automated surveillance systems that can harness those data. Barring laboratory confirmation of the diagnosis, a doctor's diagnosis and prescription may be considered as the next best specific clinical indicator of flu activity. Because “Pharmacy data provide insight into a clinician's treatment focus and might more accurately represent a patient's true condition” [14] a better specificity associated with prescription sales data might alleviate concerns surrounding ILI based data from the Google system [6].

Therefore prescription sales data might serve as a better tool of syndromic surveillance compared to Google surveillance system.

We have discussed some of the barriers to implementation already but despite sufficient proof of effectiveness there is reticence towards the use of prescription sales data as a syndromic surveillance system. We believe that these barriers are surmountable.

One such measure could be to create public private partnerships as already demonstrated by the Rhode Island model. As Electronic Medical Records (EMR) become more common, wider data source linking might further enhance SSSs' efficiency [31].

### Limitations

There are several limitations to our study. First we have not done regional analysis of the data yet. These analyses might reveal regional biases in our patterns. Second, prescription sales data include all the prophylactic prescriptions. Therefore these data may not reflect the true nature of the disease condition. Third, prescription sales data do not cover many uninsured people. This population is an important segment of the population from influenza surveillance perspective. Fourth, though our data come from a large drug retailing pharmacy chain in the United States with a solid foot-print, they still do not represent all the prescription sales data in the country. Therefore they might miss some regional patterns. Fifth, our data do not inform about the status of the high risk population. Lastly, prescription sales data are a reflection of the consequence of the disease and therefore do not speak about its onset.

### Conclusion

Community pharmacies with large footprints might want to proactively build automated SSSs with real time or near real time reporting capabilities. Furthermore they might want to explore the possibility of building predictive models around their large data sets [17]. These activities along with forming partnerships with public health agencies is another model which might contribute to syndromic surveillance and therefore to public health and safety.
